# Effects of Nitrogen Fertilizer Types and Planting Density on the Yield and Nitrogen Use Efficiency of Salt-Tolerant Rice Under Salt Stress Conditions

**DOI:** 10.3390/plants14040501

**Published:** 2025-02-07

**Authors:** Tingcheng Zhao, Jianbo Wang, Rongyi Li, Pengfei Zhang, Xiayu Guo, Yucheng Qi, Yusheng Li, Shenghai Cheng, Junchao Ji, Aibin He, Zhiyong Ai

**Affiliations:** 1 School of Breeding and Multiplication, Sanya Institute of Breeding and Multiplication, Hainan University, Sanya 572000, China; 17634021231@163.com (T.Z.); wangjianb1999@163.com (J.W.); 18790676736@163.com (R.L.); 18738491817@163.com (P.Z.); 22220951310084@hainanu.edu.cn (Y.Q.); yushengli@hainanu.edu.cn (Y.L.); csh17793665776@163.com (S.C.); 17331066936@163.com (J.J.); 2 National Innovation Center of Saline-Alkali Tolerant Ricin Sanya, Sanya 572000, China; wanilybao@163.com; 3 Hunan Hybrid Rice Research Center, Changsha 410125, China

**Keywords:** nitrogen forms, nitrogen use efficiency, planting density, salt stress, salt-tolerant rice

## Abstract

Soil salinization poses a serious threat to global food security, as high Na^+^ contents in soils hinder nitrogen use efficiency (NUE), affecting the growth and yield of crop plants. The present study aims to explore the effects of different nitrogen fertilizer types viz., NO_3_^−^ (N1) and NH_4_^+^ (N2) and planting densities, viz., D1: 30 × 10 cm, D2: 20 × 20 cm, and D3: 30 × 20 cm, on growth and development, nitrogen absorption and utilization, and yield formation. The salt-tolerant rice variety ‘Jingliangyou 3261’ was exposed to 0.3% salt irrigation water. Results revealed that N2 substantially improved the rice yield by increasing the number of effective panicles and the rate of grain-setting compared to N1. In addition, the N2 also increased leaf chlorophyll content, dry matter accumulation, antioxidant enzyme activity such as superoxide dismutase, peroxidase, and catalase activity and reduced the content of malondialdehyde. In comparison with N1, the N2 treatment resulted in an increase of 12.21%, 31.89%, and 37.53% in total nitrogen accumulation, nitrogen recovery efficiency (NRE), and nitrogen agronomic efficiency (NAE), respectively. This increase can be attributed to enhanced leaf nitrogen metabolic enzyme activity, including nitrate reductase and glutamine synthetase, and a more robust root system. Under N1 and N2 conditions, compared to D3, D1 resulted in an increase in the number of tillers but decreased the percentage of productive tillers, the grains per panicle, the grain-filling rate, and the thousand-grain weight, thereby reducing yield. Additionally, the D3 treatment also significantly improved NRE and NAE compared to the D1 treatment. Therefore, the rational selection of nitrogen fertilizer type (N2) and planting density (D3) is crucial for improving the yield and nitrogen use efficiency of salt-tolerant rice. This would broaden the scope of agricultural solutions for saline soils, potentially improving food security in regions where soil salinization is a widespread issue.

## 1. Introduction

Global climate change and unsustainable land use have made soil salinization a major environmental factor restricting agricultural production. Currently, more than 833 million hectares of land are affected by salinization worldwide [1], with over 10% of farmland impacted, posing a serious threat to global food security [2]. Rice (*Oryza sativa* L.), as one of the most important food crops in the world, is particularly vulnerable to salt stress, which significantly reduces its yield and quality [3,4]. High salt concentrations cause water imbalance, ion toxicity, and osmotic stress in plants, adversely affecting their growth and development [5,6].

Developing salt-tolerant rice varieties is a key strategy to enhance rice yield under salt stress conditions [7], as the yield reduction of salt-tolerant rice varieties is significantly lower than that of salt-sensitive rice varieties [8]. Salt-tolerant rice varieties are capable of maintaining normal physiological mechanisms, including ionic homeostasis and osmotic adjustments, as well as growth and development under saline conditions [9,10,11]. Additionally, salt-tolerant varieties have improved antioxidant defense systems to eliminate reactive oxygen species, thus protecting cells from oxidative damage [10]. Under salt stress conditions, salt-tolerant rice varieties exhibit superior agronomic traits, such as plant height, tiller number, and aboveground dry biomass, with significantly lower declines compared to salt-sensitive varieties [8]. Although salt-tolerant rice achieves higher yields, however, overall rice production is still reduced [12]. Therefore, implementing effective cultivation practices in the future could further enhance the yield of salt-tolerant rice under salt stress conditions.

Nitrogen (N) is one of the essential nutrients for plant growth and has a significant impact on the development, growth, and yield formation of crops [13]. Under salt stress conditions, the absorption and utilization efficiency of N by plants is limited, which in turn affects growth and yield [14]. Previous studies have primarily focused on increasing N application to improve the salt tolerance of rice. For example, Chen et al. [15] found that the highest rice yield was achieved with 300 kg N hm^−2^, while excess N application did not promote further yield increases. At varying salinity levels, an insufficient N supply negatively impacts the morphological and physiological characteristics of rice roots, significantly inhibiting leaf photosynthesis and ultimately reducing rice yield [15]. In addition, optimizing planting density and fertilizer management is a crucial strategy for improving rice yield under salt stress conditions. For example, with N application rates below 300 kg N hm^−2^, reducing planting density can enhance rice yield and improve grain quality [16]. Furthermore, Guo et al. [17] reported an improvement in rice yield at 0–180 kg N hm^−2^ with the increase in planting density. However, the yield was the highest at 150 kg N hm^−2^ and then decreased with increased N application. Zhu et al. [18] confirmed that reducing N fertilizer use while increasing planting density can enhance N absorption and accumulation. However, there has been limited research on the effects of varying planting densities under different N forms on rice yield, as well as N absorption and utilization efficiency.

N exists in the soil in various forms, including ammonium N (NH_4_^+^), nitrate N (NO_3_^−^), and organic N [14]. Plant roots mainly absorb N in inorganic and organic forms, with inorganic N primarily being absorbed and utilized in the form of NH_4_^+^ and NO_3_^−^ [15,19]. The utilization efficiency of different N forms by plants varies, and their effectiveness is influenced by salt stress. For instance, Nathawat et al. [20] demonstrated that, compared to NH_4_^+^, NO_3_^−^ significantly improved the salt tolerance of mustard (*Brassica juncea*), with a marked increase in biomass. Sehar et al. [21] found that supplying NH_4_^+^ increased the biomass of wheat under salt stress and enhanced its osmotic stress resistance. Previous studies have focused on the optimization of N application rates to improve salt stress tolerance in plants; however, few reports are available on the application of different types of N fertilizers in integration with varying planting densities to improve the growth, yield, and nitrogen use efficiency (NUE) in salt-tolerant rice. Therefore, the present study was conducted to assess the effects of different N fertilizer types and planting density on the morpho-physiological attributes, yield formation and NUE of salt-tolerant rice under salt stress conditions. The findings of this study are important in order to provide a scientific foundation for N management in salt-tolerant rice, aiming to enhance its yield and stability in saline conditions.

## 2. Materials and Methods

### 2.1. Experimental Conditions and Details

The field experiment was conducted from April to August 2024 at the experimental base at the National Salt-Tolerant Rice Technology Innovation Center in Leyi Village, Hainan Province (108.90′ E, 18.44′ N). The experimental treatments were arranged in a split-plot design with different forms of N fertilizers (NO_3_^−^ (in the form of calcium nitrate, with a N content of 11%) (N1) and NH_4_^+^ (in the form of ammonium sulfate, with a N content of 21%) (N2) being assigned to main plots with three different transplanting densities: D1 (30 cm × 10 cm, 332,000 hills per hm^2^); D2 (20 cm × 20 cm, 250,000 hills per hm^2^); and D3 (30 cm × 20 cm, 167,000 hills per hm^2^); two seedlings of a salt-tolerant rice variety i.e., ‘Jingliangyou’, per hill were assigned to sub-plots in triplicate. The salt-tolerant rice variety ‘Jingliangyou3261’ can be found at this link: https://www.ricedata.cn/variety/varis/631150.htm (accessed on 1 January 2022). The total amount of nitrogen (N) fertilizer was fixed at 150 kg hm^−2^, according to the following formula: basal fertilizer:tillering fertilizer:panicle fertilizer:grain fertilizer = 4:3:2:1. Each treatment uniformly used slow-release urea as the basal fertilizer (slow-release urea was polyurethane resin coated slow-release urea N: 44%; the slow-release period was 60 d). The different nitrogen fertilizer types (NH_4_^+^ and NO_3_^−^) were employed as topdressing fertilizers (comprising tillering and panicle fertilizer). Moreover, the present study also established a treatment without fertilization, with the aim of calculating NUE. The sub-plot size was 15 m^2^. A simulated saline stress environment was created through irrigation with 0.3% saltwater. Saline irrigation was applied 14 days after the tillering stage. The salinity stress was maintained continuously until the maturity stage of rice. Throughout the growth period, we regularly monitored soil salinity and supplemented with saltwater as needed to ensure the consistency of salinity stress. A conductivity instrument (2266FS, Spectrum, Middleton, WI, USA) was used to measure the salinity of water, and in cases of rainfall, the excess water was drained off from the fields and irrigate again as per requirement. Phosphatic (P_2_O_5_) fertilizer was applied at 80 kg hm^−2^ as basal and potassium (K) at 100 kg hm^−2^ was divided equally between basal and panicle fertilizer.

### 2.2. Determination of Agronomic Traits and Leaf SPAD Value

At the tillering (MT), panicle differentiation (PI), heading (HS), and maturity (MS) stages, six uniformly grown plants were randomly selected from each subplot and the number of tillers and plant height were measured and recorded. Dry matter accumulation was taken at the whole growth stages, with 6 consistent plants taken from each replicate, separated into sheaths, leaves, and panicles (after heading), and oven-dried at 105 °C for 30 min and then at 85 °C until constant weight was achieved.

The SPAD values were measured at the MT, PI, and HS stages, using a portable chlorophyll meter (SPAD-502 PLUS, Konica Minolta, Tokyo, Japan). Four uniform plants were selected from each replicate, fully expanded leaves were measured during the MT stage, and sword leaves were measured after panicle differentiation, avoiding the leaf veins, with three measurements taken on the upper, middle, and lower parts of each leaf and the average value was taken. At the HS, root morphological characteristics were measured by WinRhizo-LA1600 (Regeng Instruments Inc., Quebec, QC, Canada) root analysis.

### 2.3. Na^+^ and K^+^ Content, Total N Accumulation (TNA), and N Use Efficiency (NUE)

At the HS and MS stages, various parts of the plants were dried and ground into a powder using a sample grinder. Then, 0.3 g of the powder was microwave-digested using H_2_O_2_-H_2_SO_4_. The Kjeldahl method was used to determine the total N content in the samples. The total N accumulation and N use efficiency were calculated according to Guo et al. [17]. The Na^+^ and K^+^ contents of each group were determined using an inductively coupled plasma atomic emission spectrometer (ICP-OES) (IRIS Intrepid II XSP, Thermo, Waltham, MA, USA). N partial factor productivity (PFP_N_) (kg kg^−1^) = rice yield in each treatment (kg hm^−2^)/N fertilization application (kg hm^−2^). N agronomic efficiency (NAE) (kg kg^−1^) = (crop yield in treatment—no fertilizer application treatment) (kg hm^−2^)/nitrogen fertilizer applied (kg hm^−2^). N recovery efficiency (NRE) (%) = (total N accumulation in treatment—no fertilizer application treatment) (kg hm^−2^)/nitrogen fertilizer applied (kg hm^−2^).

### 2.4. Determination of Activities of N Metabolizing Enzymes, Antioxidant Enzymes, and Malondialdehyde (MDA) Content

At the MT, PI, and HS stages, rice leaf samples were cut and carefully stored at −80 °C in an ultra-low temperature freezer. The activities of N metabolizing enzymes such as nitrate reductase (NR) and glutamine synthetase (GS), as well as antioxidant enzymes including superoxide dismutase (SOD), peroxidase (POD), catalase (CAT), and MDA content, were determined according to the instructions of the kits produced by Beijing Solei Bao Technology Co., Ltd., Beijing, China. In brief, during the measurement, 0.1 g of the sample was accurately weighed from a self-sealing bag and placed in a tube with a capacity of 2 mL. A 1ml quantity of the extraction solution was added according to the instructions and homogenized in an ice bath. Then the activity of each enzyme was measured. The SOD activity was measured as follows: it was centrifuged at 4 °C for 10 min, the supernatant was collected, and it was mixed with other working solutions. The mixture was incubated in a water bath at 37 °C for 30 min, and then the supernatant was transferred to a 96-well plate. The absorbance of the samples was measured at 450 nm using a multi-functional microplate reader (SFS-C00002-001 Spark, Männedorf, Switzerland). The activity was expressed as the amount of enzyme required to inhibit the reduction of nitroblue tetrazolium by 50% per gram of fresh weight. The POD activity was measured as follows: the sample was centrifuged at 4 °C for 10 min, the supernatant was collected, and it was transferred to a 96-well plate. The other working solutions were added and mixed well. A multi-functional microplate reader (SFS-C00002-001 Spark, Switzerland) was used to measure the absorbance of the samples at 470 nm. The absorbance was recorded at 30 s (A1) and at 1 min and 30 s (A2), and the change in absorbance was calculated as ΔA = A2 − A1. One enzyme activity unit is defined as the amount of enzyme that causes a change in absorbance at 470 nm of 0.01 per minute in each milliliter of the reaction system per gram of tissue. The CAT activity was measured as follows: the sample was centrifuged at 4 °C for 10 min, the supernatant was collected, and it was transferred to a 96-well plate. Subsequently, the other working solutions were added and mixed well. A multi-functional microplate reader (SFS-C00002-001 Spark, Switzerland) was used to measure the initial absorbance (A1) of the samples at 240 nm and the absorbance (A2) after 1 min. The difference in absorbance was calculated as ΔA = A1 − A2. One enzyme activity unit is defined as the amount of enzyme that catalyzes the degradation of 1 μmol H₂O₂ per minute in the reaction system per gram of tissue. The NR activity was measured as follows: the sample was centrifuged at 4 °C for 10 min, the supernatant was collected, and it was mixed with other working solutions. A multi-functional microplate reader (SFS-C00002-001 Spark, Switzerland) was used to measure the initial absorbance (A1) at −340 nm. Then, the reaction was incubated at 25 °C for 30 min and measure the absorbance (A2) again. The change in absorbance was calculated for the test tube as ΔA_test tube = A1_test tube − A2_test tube, and for the blank tube as ΔA blank tube = A1_blank tube − A2_blank tube. Finally, the net change in absorbance was calculated as ΔA = ΔA_test tube − ΔA_blank tube. One NR activity unit is defined as the amount of enzyme that consumes 1 μmol of NADH per hour per gram of sample. The GS activity was measured as follows: the sample was centrifuged at 4 °C for 10 min, the supernatant was collected, and it was mixed with other working solutions. After mixing, it was allowed to stand for 10 min and then was centrifuged at room temperature for 10 min and the supernatant was transferred to a 96-well plate. Using a multi-functional microplate reader (SFS-C00002-001 Spark, Switzerland), the absorbance (A) was measured at 540 nm. The change in absorbance was calculated as ΔA = A_test − A_control. One enzyme activity unit is defined as the amount of enzyme that causes a change in absorbance at 540 nm of 0.01 per minute in the reaction system per gram of tissue. Leaf samples (0.5 g) were homogenized in 5 mL of 5% trichloroacetic acid. The method for MDA determination is based on Li et al. [2]. In brief, the homogenate was centrifuged at 4000× *g* for 10 min at 25 °C, and 3 mL of 2-thiobarbituric acid in 20% trichloroacetic acid was added to a 2 mL aliquot of the supernatant. The mixture was heated at 98 °C for 10 min and cooled rapidly in an ice bath. After centrifugation at 4000× *g* for 10 min, the absorbance was recorded at 532 nm. Measurements were corrected for non-specific turbidity by subtracting the absorbance at 600 nm. MDA concentration was determined by the extinction coefficient MDA.

### 2.5. Determination of Rice Yield and Yield Attributes

At MS, three investigation points in each experimental subplot were marked, and 20 effective panicles were investigated at each point for the average number of effective panicles. The percentage of productive tillers was calculated by dividing the number of effective panicles by the number of tillers at the MT stage. Moreover, based on the average number of effective panicles, five consistent panicles were taken, one sample per subplot, and the total number of grains per panicle, seed-setting rate, and thousand-grain weight were measured. For yield assessment, 6 square meters from each subplot were harvested, and after removing the empty grains, the samples were weighed and converted to yield based on a standard moisture content of 13.5%.

### 2.6. Statistical Analysis

Two-way analysis of variance (ANOVA) was conducted on the collected data using SPSS 19.0 software (SPSS, Inc., Chicago, IL, USA). The differences amongst the treatment means of each experiment were determined using the least significant difference (LSD) test at a significance level of 0.05. All graphs were created using Origin 9.0 (OriginLab Corp., Northampton, MA, USA).

## 3. Results

### 3.1. Rice Yield and Yield Components

Different N fertilizer types and planting densities significantly affect the yield and yield component of rice (Table 1). Under N1 conditions, the yield of D1, D2, and D3 were 7.06, 7.61, and 7.81 t hm^−2^, respectively. Compared to N1, the N2 led to a 5.32, 1.15, and 9.21% increase in the number of effective panicles, seed-setting rate, and grain yield, respectively. However, no significant difference was noticed in the number of grains per panicle and the thousand-grain weight between the N1 and N2 treatments. Regarding planting density, no significant difference in yield was noticed between the D3 and D2 treatments, whereas D3 led to an increase in grain yield by 10.62% and 5.03% under N1 and N2 conditions, respectively, compared to D1. Furthermore, under N1 and N2 conditions, the number of effective panicles was the highest with the D1 treatment. Additionally, the trends in the number of grains per panicle, the rate of grain-setting, and the thousand-grain weight across all planting densities were consistent under N1 and N2 conditions, ranking from high to low as follows: D1 < D2 < D3. Under N1 and N2 conditions, no significant differences in the number of grains per panicle between D3 and D2 were noticed. However, the D3 treatment led to 12.76% and 13.39% increase in N1 and N2, respectively, as compared with D1. Moreover, the seed-setting rate and the thousand-grain weight of the D1 treatment were also substantially lower than those of the D3 treatment. Under the N1 treatment, the filled grains per plant were 1122.3, 1473.3, and 2146.7 for D1, D2, and D3, respectively. No significant differences were found between N1 and N2 for filled grains per plant or for unfilled grains per plant.

### 3.2. N Accumulation and NUE

Different forms of N fertilizer types and planting densities substantially affected N accumulation and NUE in rice (Table 2). Compared with N1, N2 increased N accumulation by 12.21%. Furthermore, N2 substantially enhanced N use efficiency, including N partial factor productivity (PFP_N_), N recovery efficiency (NRE), and N agronomic efficiency (NAE) by 9.19%, 31.89%, and 37.53%, respectively. With different N fertilizer types, significant differences in N accumulation were noted among various planting densities, with the highest in the D3 treatment, which was 4.20% and 5.34% higher than D1 under N1 and N2 conditions, respectively. Regarding PFP_N_, with N1, no significant difference between D2 and D3 plants was noticed. Nevertheless, they were 7.75% and 10.56% higher than D1 plants, respectively. In addition, under both N1 and N2 conditions, the trends of NRE and NAE were consistent among different planting densities: i.e., D1 < D2 < D3. For NRE and NAE under N1 and N2 conditions, no significant differences between the D2 and D3 treatments were noticed, but both were significantly higher than D1.

### 3.3. Agronomic Traits

**Figure 1:**
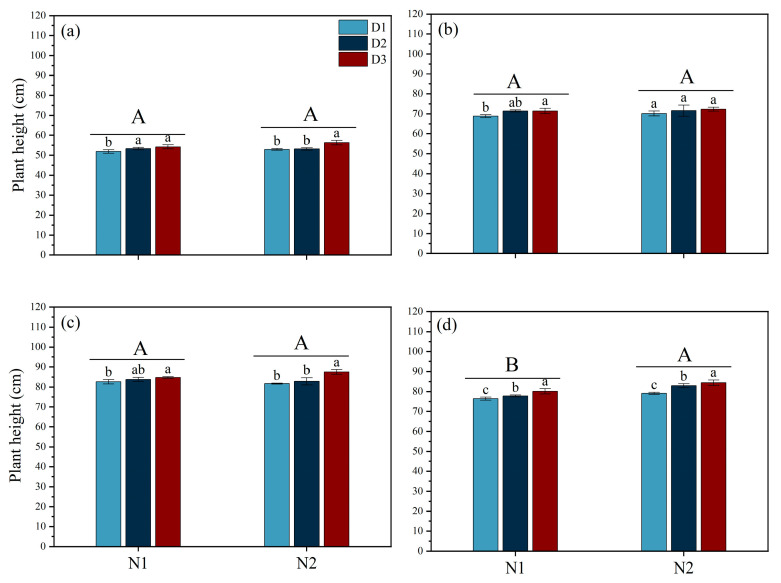
Effects of different nitrogen forms and planting density on plant height under saline conditions. Plant height during tillering stage (**a**); plant height during panicle differentiation stage (**b**); plant height during heading stage (**c**); plant height during maturity stage (**d**). Note: Different lowercase letters indicate significant differences found using the LSD test for *p* < 0.05 between planting density treatments under the same different nitrogen forms; different uppercase letters indicate significant differences found using the LSD test for *p* < 0.05 between N1 and N2. N1 and N2 represent NO_3_^−^ and NH_4_^+^ fertilizers, respectively. NO_3_^−^ and NH_4_^+^ are provided by calcium nitrate and ammonium sulfate fertilizer, respectively. D1: 30 × 10 cm; D2: 20 × 20 cm; D3: 30 × 20 cm.

Different N fertilizer types and planting densities significantly affect plant height (Figure 1). No significant differences in plant height were observed between N1 and N2 during the MT, PI, and HS stages. However, at the MS stage, plant height under N2 was significantly greater than under N1. During the MT stage, plant height under both N1 and N2 conditions was the highest in D3, significantly surpassing D1 by 4.48% and 6.39%, respectively. During the PI stage, no significant differences in plant height were observed between D1 and D2. At the HS and MS stages, plant height followed a consistent trend under both N1 and N2 treatments, ranking in the order D1 < D2 < D3. At the MS stage, D3 was significantly taller than those in D1 and D2. Under N1 conditions, D3 exceeded D1 by 4.80% and D2 by 6.74%, while under N2 conditions, D3 was taller than D1 and D2 by 3.00% and 1.81%, respectively.

**Figure 2:**
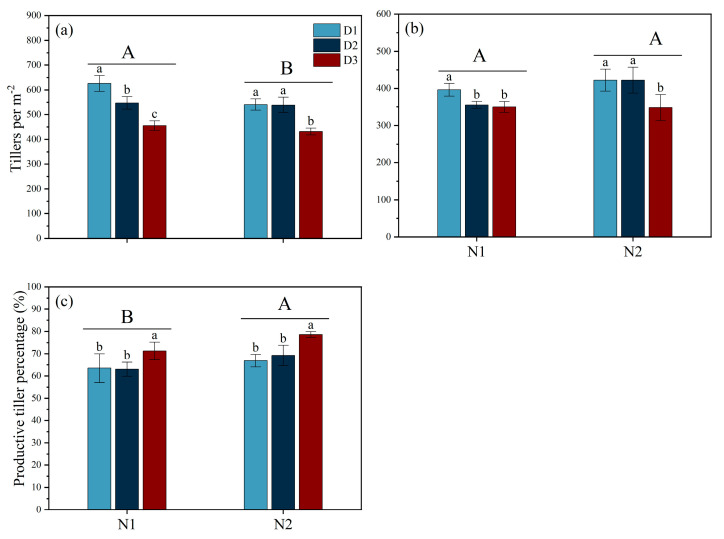
Effects of different nitrogen forms and planting density on tillers per m^−2^ and productive tiller percentage under saline conditions. Tillers per m^−2^ during tillering stage (**a**); Tillers per m^−2^ during panicle differentiation stage (**b**); productive tiller percentage (**c**). Note: Different lowercase letters indicate significant differences found using the LSD test for *p* < 0.05 between planting density treatments under the same different nitrogen forms; different uppercase letters indicate significant differences found using the LSD test for *p* < 0.05 between N1 and N2. N1 and N2 represent NO_3_^−^ and NH_4_^+^ fertilizers, respectively. NO_3_^−^ and NH_4_^+^ are provided by calcium nitrate and ammonium sulfate fertilizer, respectively. D1: 30 × 10 cm; D2: 20 × 20 cm; D3: 30 × 20 cm.

Different types of N fertilizer and planting densities significantly influenced the number of tillers per m^2^ during the MT and PI stages and the productive tiller percentage (Figure 2). During the MT stage, under N1 conditions, the tillers per D1, D2, and D3 plant were 18.7, 22.0, and 27.3, respectively. The tillers per m^2^ under the N1 treatment were significantly higher than those under the N2 treatment by 7.78% at the MT stage; however, no significant differences were observed between N1 and N2 during the PI stage. Additionally, under both N1 and N2 conditions, the tillers per m^2^ with the D3 treatment were the lowest during the MT and PI stages, significantly lower than those in D1. Moreover, during the MT, the tillers per m^2^ in D1 exceeded D3 by 27.22% and 20.21% under N1 and N2 conditions, respectively. During the PI stage, D1 was 11.68% and 17.54% higher than D3 under N1 and N2 conditions, respectively. The productive tiller percentage in the N2 treatment was significantly higher than that in the N1 group. Furthermore, under both N1 and N2 treatments, the productive tiller percentage with the D3 treatment was 12.20% and 17.51% higher than D1 and 13.04% and 13.51% higher than D2, respectively.

**Figure 3:**
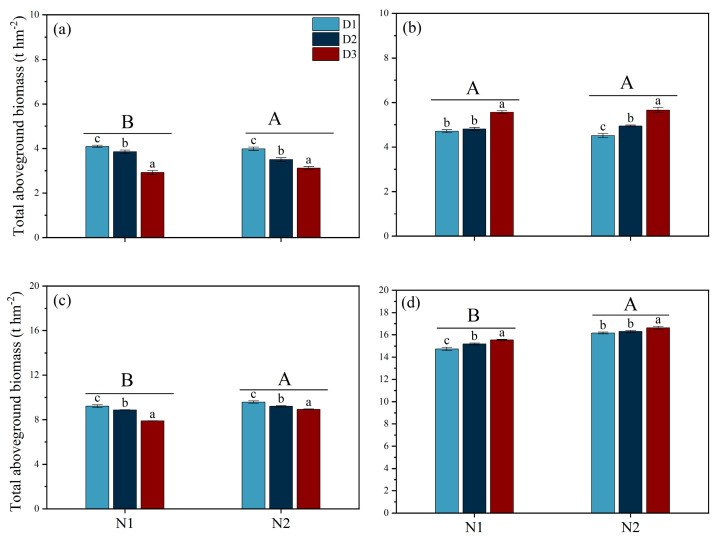
Effects of different nitrogen forms and planting density on total aboveground biomass under saline conditions. Total aboveground biomass during tillering stage (**a**); total aboveground biomass during panicle differentiation stage (**b**); total aboveground biomass during heading stage (**c**); total aboveground biomass during maturity stage (**d**). Note: Different lowercase letters indicate significant differences found using the LSD test for *p* < 0.05 between planting density treatments under the same different nitrogen forms; different uppercase letters indicate significant differences found using the LSD test for *p* < 0.05 between N1 and N2. N1 and N2 represent NO_3_^−^ and NH_4_^+^ fertilizers, respectively. NO_3_^−^ and NH_4_^+^ are provided by calcium nitrate and ammonium sulfate fertilizer, respectively. D1: 30 × 10 cm; D2: 20 × 20 cm; D3: 30 × 20 cm.

During the MT stage, under both N1 and N2 conditions, D1 exhibited the highest dry matter accumulation, significantly exceeding D2 by 6.06% and 13.88% and D3 by 40.24% and 28.08%, respectively. In contrast, during the PI stage, the trend was reversed. Under both N1 and N2 conditions, at the HS and MS stages, the trend in aboveground dry matter accumulation across planting densities was consistent under both the N1 and N2 treatments, ranking in the order D1 < D2 < D3 (Figure 3).

In addition, different forms of N fertilizer and planting densities significantly affected the root morphological indices of rice (Table 3). For instance, compared to N1, N2 significantly increased total root volume, total root length, root surface area, and average root diameter by 6.30%, 5.33%, 11.25%, and 4.23%, respectively. Under both forms of N fertilizer, the root morphological indices varied significantly among planting densities, with the highest values observed in the D3 treatment, ranking from low to high in the order D1 < D2 < D3.

### 3.4. Leaf SPAD Value

**Figure 4:**
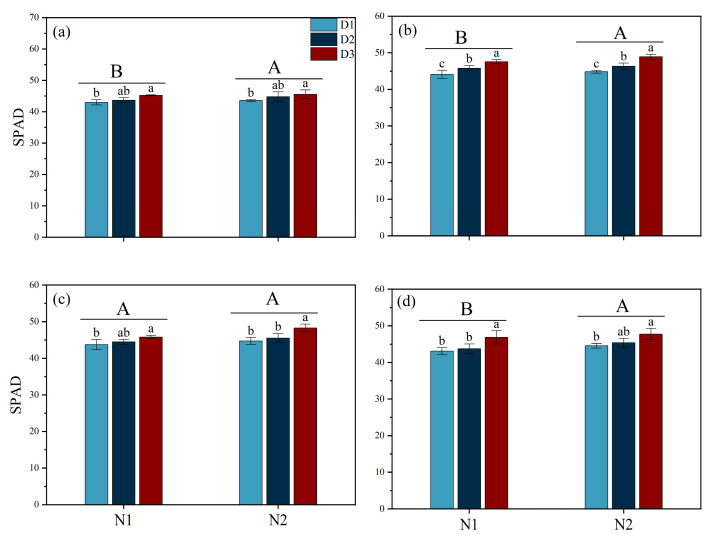
Effects of different nitrogen forms and planting density on leaf SPAD under saline conditions. SPAD during tillering stage (**a**); SPAD during panicle differentiation stage (**b**); SPAD during heading stage (**c**); SPAD during maturity stage (**d**). Note: Different lowercase letters indicate significant differences found using the LSD test for *p* < 0.05 between planting density treatments under the same different nitrogen forms; different uppercase letters indicate significant differences found using the LSD test for *p* < 0.05 between N1 and N2. N1 and N2 represent NO_3_^−^ and NH_4_^+^ fertilizers, respectively. NO_3_^−^ and NH_4_^+^ are provided by calcium nitrate and ammonium sulfate fertilizer, respectively. D1: 30 × 10 cm; D2: 20 × 20 cm; D3: 30 × 20 cm.

Different N fertilizer forms and planting densities significantly influenced the SPAD values of rice at various growth stages (Figure 4). During the MT, PI, and MS stages, the SPAD values under N1 were 1.51%, 3.34%, and 2.93% higher than those under N2, respectively. During the MT stage, under both N1 and N2 conditions, the SPAD values of leaves in the D3 treatment were 5.20% and 4.99% higher than those of D1, respectively. During the PI stage, under both N1 and N2 conditions, significant differences were observed among all planting density treatments, with D3 showing the highest SPAD values.

### 3.5. Antioxidant Enzyme Activity

**Figure 5:**
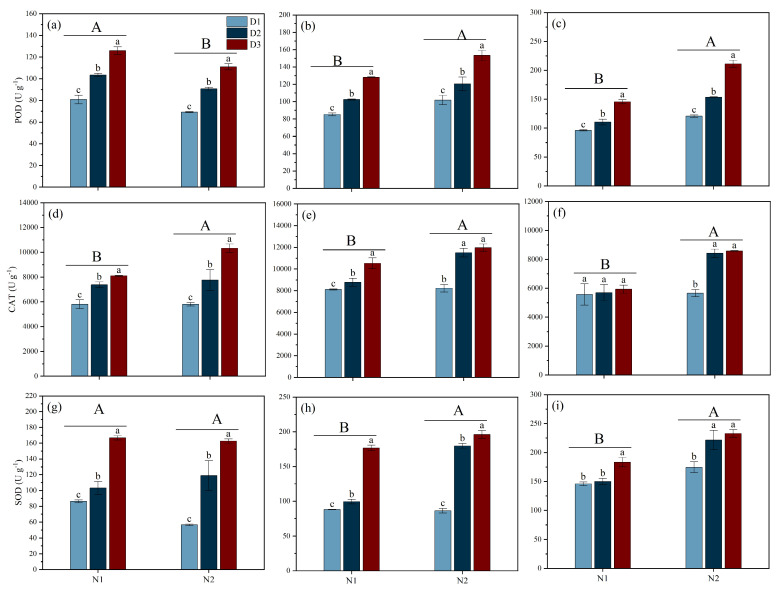
Effects of different nitrogen forms and planting density on leaf antioxidant enzyme activity under saline conditions. SOD activity during tillering stage (**a**); SOD activity during panicle differentiation stage (**b**); SOD activity during heading stage (**c**); POD activity during tillering stage (**d**); POD activity during panicle differentiation stage (**e**); POD activity during heading stage (**f**); CAT activity during tillering stage (**g**); CAT activity during panicle differentiation stage (**h**); CAT activity during heading stage (**i**). Note: Different lowercase letters indicate significant differences found using the LSD test for *p* < 0.05 between planting density treatments under the same different nitrogen forms; different uppercase letters indicate significant differences found using the LSD test for *p* < 0.05 between N1 and N2. N1 and N2 represent NO_3_^−^ and NH_4_^+^ fertilizers, respectively. NO_3_^−^ and NH_4_^+^ are provided by calcium nitrate and ammonium sulfate fertilizer, respectively. D1: 30 × 10 cm; D2: 20 × 20 cm; D3: 30 × 20 cm. SOD, superoxide dismutase; POD, peroxidase; CAT, catalase.

Different N fertilizer types and planting densities significantly affected the antioxidant enzyme activity in rice leaves at various stages (Figure 5). For POD activity, during the MT stage, N1 was significantly higher than N2. However, during the PI and HS stages, N1 was significantly lower than N2, with reductions of 19.00% and 37.87%, respectively. Additionally, under N1 and N2 conditions, the POD enzyme activity at the MT, PI, and HS stages showed a consistent trend across all planting densities, ranking from low to high in the order of D1 < D2 < D3.

The N2 treatment increased CAT activity more than the N1 treatment by 12.32%, 15.69%, and 31.82% at the MT, PI, and HS stages, respectively, whereas the CAT activity in D3 was increased by 33.06% and 9.97% in D2, and 39.59% and 77.89% in D1 under N1 and N2 conditions, respectively. During the PI stage, under N1 treatment, D3 exhibited the highest CAT activity. However, under N2 conditions, the CAT activity in D2 and D3 was 39.90% and 45.45% higher than that in D1, respectively. Moreover, at the HS stage, no significant differences in CAT activity were observed among planting densities under N1 treatment. In contrast, under N2 conditions, the CAT activity in D1 was 48.72% and 51.60% lower than that in D2 and D3, respectively.

Regarding SOD activity, there was no significant difference between N1 and N2 at the MT stage. However, N1 was significantly lower than N2 at the PI and HS stages. Additionally, during the MT and PI stages, significant differences in SOD activity were observed among all planting density treatments, with a consistent trend ranking from low to high in the order D1 < D2 < D3. During the HS stage, under N1 conditions, no significant differences in SOD activity were found between D1 and D2; however, both were significantly lower than D3 by 25.86% and 22.37%, respectively. Under N2 conditions, D1 exhibited the lowest SOD activity, significantly lower than D2 and D3 by 27.09% and 33.54%, respectively.

**Figure 6:**
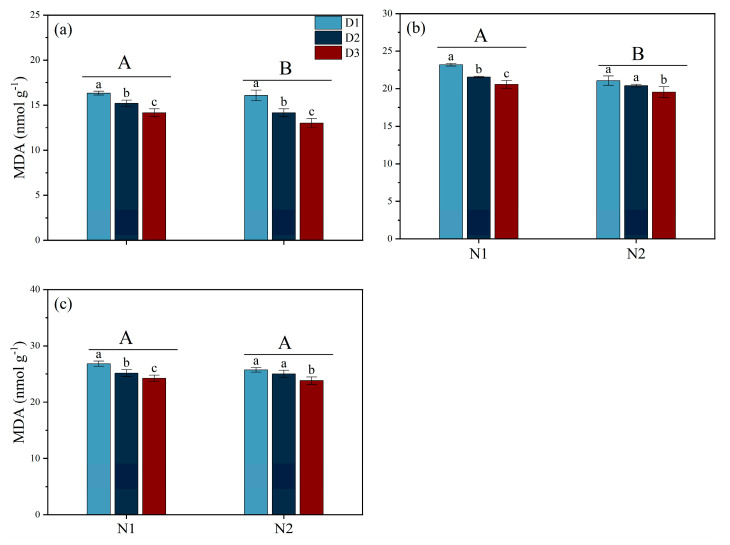
Effects of different nitrogen forms and planting density on leaf malondialdehyde (MDA) content under saline conditions. MDA content during tillering stage (**a**); MDA content during panicle differentiation stage (**b**); MDA content during heading stage (**c**). Note: Different lowercase letters indicate significant differences found using the LSD test for *p* < 0.05 between planting density treatments under the same different nitrogen forms; different uppercase letters indicate significant differences found using the LSD test for *p* < 0.05 between N1 and N2. N1 and N2 represent NO_3_^−^ and NH_4_^+^ fertilizers, respectively. NO_3_^−^ and NH_4_^+^ are provided by calcium nitrate and ammonium sulfate fertilizer, respectively. D1: 30 × 10 cm; D2: 20 × 20 cm; D3: 30 × 20 cm.

In addition, during the MT and PI stages, the MDA content in the leaves of N2 plants was significantly lower than in those receiving the N1 treatment, and there was no significant difference between the N1 and N2 treatments at the HS stage. In addition, under different N forms, the trend of leaf MDA content was consistent at various stages, ranking from high to low as follows: D1 > D2 > D3 (Figure 6).

### 3.6. Leaf Nitrate Reductase (NR) and Glutamine Synthetase (GS) Activity

**Figure 7:**
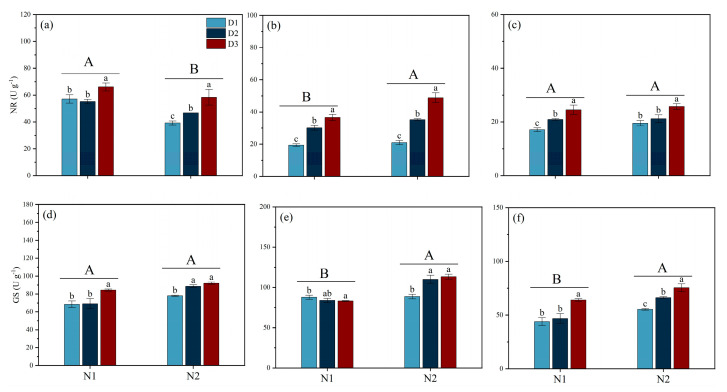
Effects of different nitrogen forms and planting density on NR and GS activity under saline conditions. NR activity during tillering stage (**a**); NR activity during panicle differentiation stage (**b**); NR activity during heading stage (**c**); GS activity during tillering stage (**d**); GS activity during panicle differentiation stage (**e**); GS activity during heading stage (**f**). Note: Different lowercase letters indicate significant differences found using the LSD test for *p* < 0.05 between planting density treatments under the same different nitrogen forms; different uppercase letters indicate significant differences found using the LSD test for *p* < 0.05 between N1 and N2. NO_3_^−^ and NH_4_^+^ are provided by calcium nitrate and ammonium sulfate fertilizer, respectively. D1: 30 × 10 cm; D2: 20 × 20 cm; D3: 30 × 20 cm. GS, glutamine synthetase; NR, nitrate reductase.

Different N fertilizer types and planting densities significantly influenced the activities of N metabolic enzymes NR and GS at various growth stages (Figure 7). For NR activity, activity under N1 conditions was significantly higher than under N2 conditions at the MT stage. However, at the PI stage, the NR activity under N1 was significantly lower than under N2, with a reduction of 21.96%. Under N1 treatment, there was no significant difference in NR activity between the D1 and D2 treatments at the MT stage; however, both were 15.53% and 19.75% lower than D3, respectively. Under N2 conditions, the NR activity in D2 and D3 was increased by 18.82% and 48.84% compared to D1. During the PI and HS stages, under both N1 and N2 conditions, NR enzyme activity followed a consistent trend across planting densities, ranking from low to high as D1 < D2 < D3.

For GS activity, during the MT, PI, and HS stages, N2 had the highest activity, exceeding N1 by 16.52%, 22.49%, and 27.42%, respectively. Under N1 conditions, during the MT stage, GS activity was highest in D3, exceeding that in D2 and D1 by 23.36% and 22.29%, respectively. Under N2 treatment, there was no significant difference in GS activity between D2 and D3, but both were 13.70% and 18.10% higher than D1, respectively. During the PI stage, under N1 conditions, the trend in GS activity was the opposite of that observed during the MT stage, with the D1 treatment showing the highest activity. During the HS stage, under both N1 and N2 conditions, the GS activity in the D1 and D2 treatments was significantly lower than in D3.

### 3.7. Sodium (Na^+^) and Potassium Ions (K^+^) Contents and Their Ratio

**Figure 8:**
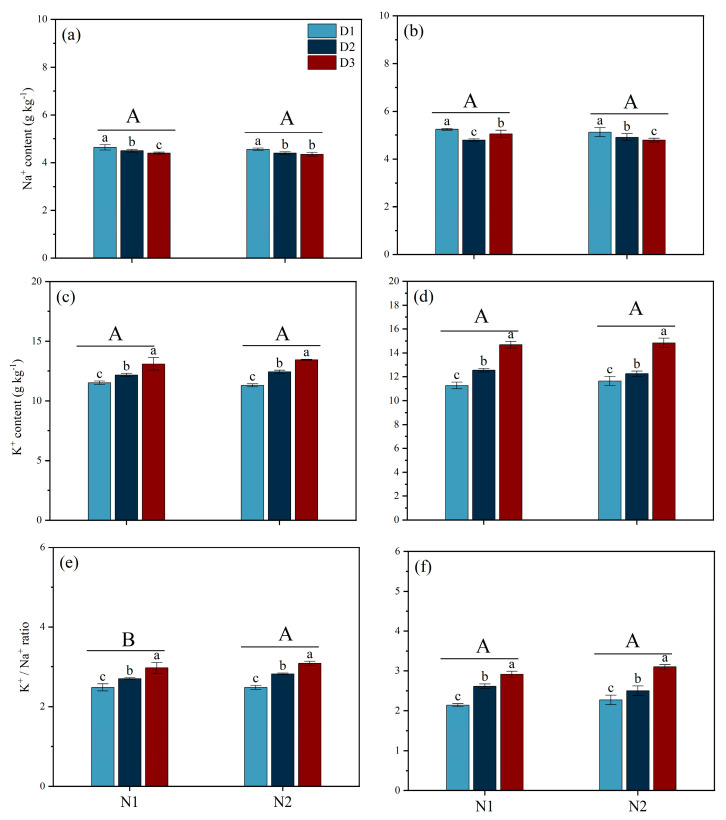
Effects of different nitrogen forms and planting density on flag and stem leaf sodium (Na^+^) and potassium ions (K^+^) and their ratio during heading stage under saline conditions. Note: Leaf Na^+^ content (**a**); stem Na^+^ content (**b**); leaf K^+^ content (**c**); stem K^+^ content (**d**); leaf K^+^/Na^+^ ratio (**e**); stem K^+^/Na^+^ ratio (**f**). Different lowercase letters indicate significant differences found using the LSD test for *p* < 0.05 between planting density treatments under the same different nitrogen forms; different uppercase letters indicate significant differences found using the LSD test for *p* < 0.05 between N1 and N2. N1 and N2 represent NO_3_^−^ and NH_4_^+^ fertilizers, respectively. NO_3_^−^ and NH_4_^+^ are provided by calcium nitrate and ammonium sulfate fertilizer, respectively. D1: 30 × 10 cm; D2: 20 × 20 cm; D3: 30 × 20 cm.

Different forms of N fertilizer and planting densities have a significant impact on Na^+^ and K^+^ ion content and their ratios in the leaves and stems at the HS stage (Figure 8). Under both N1 and N2 conditions, the Na^+^ level in the leaves of the D3 treatment was significantly lower than that of the D1 treatment by 5.17% and 4.61%, respectively, and the Na^+^ content in the stems of the D3 treatment was significantly lower than that of the D1 treatment by 3.81% and 6.63%, respectively. For K^+^ content and K^+^/Na+ ratio, with different N forms, the trends in the stems and leaves were consistent, ranking from high to low in the order D3 > D2 > D1. Moreover, under both N1 and N2 conditions, compared with D1, the K^+^ content in the leaves of the D3 treatment increased by 13.81% and 19.03%, respectively, whereas the K^+^ content in the stems increased by 30.37% and 27.49%, respectively.

## 4. Discussion

### 4.1. Effects of Different N Forms and Planting Density on Yield of Salt-Tolerant

Nutrient deficiency under salt stress often leads to a decrease in rice grain yield. In addition to breeding salt-tolerant rice varieties, the use of appropriate N fertilizer management and planting density are key strategies to mitigate the negative effects of salinity [14,15].

Previous studies indicate that the absorption and utilization of N by plants is significantly limited, thus affecting their growth, development, and yield under saline conditions. The results of the present study showed that the yield of rice when NH_4_^+^ (N2) is used as a basal fertilizer is significantly higher than that under NO_3_^−^ (N1), with the main reason being the significant increase in the number of effective panicles and the grain-filling rate (Table 1). Our findings are inconsistent with those of Yan et al. [22], who found that increasing the number of effective panicles m^−2^ is crucial for improving grain yield in saline–alkali rice soils. The number of tillers in cereal crops is highly sensitive to N, and studies have shown that NH_4_^+^ is beneficial for increasing the number of effective panicles, while NO_3_^−^ is beneficial for increasing the number of grains per panicle [23]. NH_4_^+^, as a N source that plants can directly absorb and utilize, may provide a more effective N supply in salt stress environments, promoting the growth and development of rice [14]. Additionally, the N2 treatment maintained the chlorophyll content in the leaves during the later stages of growth and improved the total aboveground biomass during the HS stage, which possibly contributed to an increase in the number of effective panicles and the rate of grain-setting (Figure 3 and Figure 4). The superior performance of NH_4_^+^ may be attributed to its metabolic and utilization characteristics within the plant. The application of NH_4_^+^ can reduce the efflux of K^+^ while limiting the accumulation and transport of Na^+^ under salt stress conditions, which helps maintain a favorable K^+^/Na^+^ balance in order to improve salt tolerance in plants [19]. These results were in agreement with those of our study. We found that N2 treatment increased the K^+^ content in leaves and thus decreased the K^+^/Na^+^ ratio. Furthermore, our study found that after the PI stage, the antioxidant enzyme activity in the N2 treatment group was significantly higher than in the N1 treatment group, and the MDA content was lower than in the N1 treatment group (Figure 5 and Figure 6). Previous studies have shown that NH_4_^+^ is more suitable as a N source under salt stress because NO_3_^−^ can be partially replaced by chloride during root absorption, while NH_4_^+^ can enhance the osmotic adjustment ability of corn to salt by accumulating inorganic solutes, thus promoting its tolerance under salt stress [24]. Li et al. [23] found that NH_4_^+^ can alleviate the damage of salt stress and reactive oxygen species (ROS) to plants. Additionally, with the application of NH_4_^+^, the content of total phenolic compounds and flavonoids and antioxidant enzyme activity was increased in ryegrass under salt stress conditions [23]. The application of NH_4_^+^ can reduce damage caused by ROS by promoting the activity of antioxidant enzymes, which help decompose ROS [25]. Additionally, the phenolic compounds produced by plants are strong antioxidants, and their metabolism assists plants in coping with nutritional imbalances caused by oxidative stress.

Guo et al. [17] found that under salt stress conditions, increasing planting density within the range of 0–150 kg hm^−2^ of fertilizer application can increase the number of effective panicles. This study is consistent with our findings, which showed that although high density could increase tiller density, it also reduced grains per panicle, the grain-filling rate, and the thousand-grain weight, thereby reducing yield (Table 1 and Figure 2). Furthermore, our study found that under salt stress conditions, increasing density also reduced productive tiller percentage, which may be related to intensified competition among rice plants under high-density planting conditions [26]. This competition not only affects the tillering ability of rice but may also lead to uneven nutrient distribution of photo-assimilates [27]. Additionally, excessive tillers consume a large amount of nutrients, reducing the nutrient resources for other tillers and leading to a decrease in the number of grains per panicle, the rate of grain-setting, and the thousand-grain weight [28]. Moreover, lower planting density may also help improve light conditions and ventilation for rice, reduce the occurrence and spread of diseases, and possibly increase the stress resistance of rice [29,30,31], which is consistent with our findings, which show that in low-density conditions, the stress-resistant enzyme activity in rice leaves is significantly enhanced, and the MDA content is reduced. Therefore, optimization of planting density based on specific soil conditions, climate environment, rice varieties, and other factors is important to achieve high yield and quality in salt-tolerant rice.

### 4.2. Effects of Different N Forms and Planting Densities on the N Absorption and Utilization Efficiency of Salt-Tolerant Rice

This study also found that N2 exhibited a clear advantage in N absorption and utilization, whereas N2 demonstrated greater total nitrogen accumulation and NUE. In general, plants need to enhance their N absorption and utilization to sustain normal physiological processes, growth, and development under salt stress conditions [15]. NH_4_^+^, due to its direct availability and unique metabolic characteristics within plants, may help promote N absorption and utilization in rice, thereby enhancing its salt tolerance [32]. The results demonstrated that the activities of NR and GS in N2 treatment groups were significantly higher than those in N2 treatment groups during the PI stage (Figure 7). These findings imply that elevated nitrogen-related enzyme activities promoted the transformation of nitrogen, which consequently increased NUE. Furthermore, our study results indicate that root development with the NH_4_^+^ treatment is superior to that with NO_3_^−^ treatment (Table 3). In terms of root development, supplying NO_3_^−^ may result in poor root development, while supplying NH_4_^+^ has a smaller impact on the root system of rice [33]. Under osmotic stress conditions, rice seedlings supplied with NH_4_^+^ have higher aquaporin activity, a greater number of lateral roots, and a lower density of aerenchyma, thus giving their roots a higher water absorption capacity and enhancing their tolerance to osmotic stress [33].

It has been reported that in rice production under normal conditions, high planting density is recommended to reduce N input [34,35]. However, compared to rice grown in non-saline fields, salt-affected rice requires higher N levels to enhance its salt tolerance. This is primarily because salinity negatively impacts the tillering ability of rice. Therefore, to ensure a reasonable and adequate population structure, both the fertilization rate (300 kg N hm^−2^) and planting density must be increased for rice grown under saline–sodic conditions [36]. However, due to ion competition, which can inhibit nutrient absorption and lead to significant nutrient loss, N use efficiency tends to be low [16]. In our study, using NH_4_^+^-based fertilizers under low-density conditions (30 × 20) improved the salt tolerance of rice and promoted the accumulation of N in various parts. Additionally, low-density conditions facilitated the translocation of nutrients to the grains, increasing the proportion of N accumulation in the grains and thereby raising the NUE. At the same time, a lower planting density also improved the N absorption and utilization efficiency of rice. This is because a lower planting density reduces competition and shading among plants, allowing each plant to receive more light and access to nutritional space, which benefits root growth and development, as well as the absorption and utilization of soil nutrients [26]. Therefore, under saline–sodic conditions, providing an adequate amount of fertilizer and selecting the appropriate type of N fertilizer and planting density can enhance nutrient absorption and utilization that may ensures a synergistic increase in N use efficiency and yield.

## 5. Conclusions

Under salt stress conditions, the NH_4_^+^ treatment significantly increased rice yield by enhancing the number of effective panicles and the grain-filling rate as well as improving chlorophyll content, dry matter accumulation, antioxidant enzyme activity, and reduced malondialdehyde content, thereby improving salt tolerance, as compared with NO_3_^−^ treatment. High planting density increased total tillers while reduced the productive tiller percentage, grains per panicle, grain-filling rate, and thousand-grain weight, thus lowering yield. The NH_4_^+^ treatment also promoted better root systems and higher N-related enzyme activity, enhancing N absorption and transformation, especially under low-density planting. Therefore, combining NH_4_^+^ fertilizer with optimum plant density, i.e., 30 cm × 20 cm, could promote the grain yield and NUE of salt-tolerant rice under saline conditions. Building upon these findings, future research endeavors should focus on expanding the scope of investigation to encompass a broader range of salt concentrations and further refine the understanding of the interplay between nitrogen fertilizer types, planting densities, and salt stress.

## Figures and Tables

**Table 1:** Effects of different nitrogen forms and planting density on yield and yield components of salt-tolerant rice under saline conditions.

Treatment	Planting Density	Grain Yield (t hm^−2^)	Effective Panicles (m^−2^)	Spikelets per Panicle (No Panicle^−1^)	Unfilled Grains per Plant	Filled Grains per Plant	Grain Filling Rate (%)	Thousand-Grain Weight (g)
N1	D1	7.06 b	396.3 a	110.5 b	188.7 c	1122.3 c	85.6 b	19.5 c
	D2	7.61 ab	344.3 b	123.9 a	232.0 b	1473.3 b	86.4 b	21.1 b
	D3	7.81 a	324.3 b	124.6 a	276.7 a	2146.7 a	88.6 a	22.4 a
	Mean	7.49 B	355.0 B	119.8 A	232.4 A	1580.8 A	87.0 B	21.0 A
N2	D1	7.95 b	411.0 a	112.0 b	189.0 c	1190.0 c	86.3 c	20.4 b
	D2	8.24 ab	372.0 b	124.0 ab	223.0 b	1617.7 b	87.9 b	20.8 b
	D3	8.35 a	338.7 c	127.0 a	261.3 a	2312.3 a	89.8 a	22.3 a
	Mean	8.18 A	373.9 A	121.0 A	224.4 A	1706.7 A	88.0 A	21.2 A

Note: Different lowercase letters indicate significant differences found using the LSD test for *p* < 0.05 between planting density treatments with the same or different nitrogen forms. Different uppercase letters indicate significant differences found with the LSD test for *p* < 0.05 between N1 and N2. N1 and N2 represent NO_3_^−^ and NH_4_^+^ fertilizers, respectively. NO_3_^−^ and NH_4_^+^ are provided by calcium nitrate and ammonium sulfate fertilizer, respectively. D1: 30 ×10 cm; D2: 20 × 20 cm; D3: 30 × 20 cm.

**Table 2:** Effects of different nitrogen forms and planting density on total nitrogen accumulation and nitrogen use efficiency of salt-tolerant rice under saline conditions.

Treatment	Planting Density	TNA (kg hm^−2^)	PFP_N_ (kg kg^−1^)	NRE (%)	NAE (kg kg^−1^)
N1	D1	143.60 ± 1.09 c	47.07 ± 0.18 b	31.18 ± 0.91 b	8.24 ± 1.72 b
	D2	146.89 ± 0.35 b	50.72 ± 2.80 a	40.15 ± 0.50 a	13.89 ± 2.99 a
	D3	149.63 ± 0.79 a	52.04 ± 1.75 a	40.97 ± 1.05 a	14.56 ± 3.16 a
	Mean	146.71B	49.94B	37.44B	12.23B
N2	D1	159.98 ± 1.20 c	52.96 ± 1.02 a	42.10 ± 1.02 b	14.13 ± 1.76 b
	D2	165.34 ± 2.45 b	54.94 ± 0.87 a	52.46 ± 1.59 a	18.11 ± 0.80 a
	D3	168.53 ± 0.47 a	55.68 ± 0.83 a	53.57 ± 1.30 a	18.21 ± 1.82 a
	Mean	164.62A	54.53A	49.38A	16.82A

Note: Different lowercase letters indicate significant differences found using the LSD test for *p* < 0.05 between planting density treatments under the same or different nitrogen forms. Different uppercase letters indicate significant differences between N1 and N2 found using the LSD test for *p* < 0.05. N1 and N2 represent NO_3_^−^ and NH_4_^+^ fertilizers, respectively. NO_3_^−^ and NH_4_^+^ are provided by calcium nitrate and ammonium sulfate fertilizer, respectively. D1: 30 × 10 cm; D2: 20 × 20 cm; D3: 30 × 20 cm. TNA, total nitrogen accumulation; PFP_N_, nitrogen partial factor productivity; NRE, nitrogen recovery efficiency; NAE, nitrogen agronomic efficiency.

**Table 3:** Effects of different nitrogen forms and planting density on roots morphology index of salt-tolerant rice under saline conditions.

Treatment	Planting Density	Total Root Volume (cm^3^)	Total Root Length (cm)	Root Superficial Area (cm^2^)	Average Root Diameter (mm)
N1	S1	29.04 ± 0.52 c	4476.45 ± 15.28 c	1053.75 ± 7.66 c	0.61 ± 0.02 c
	S2	44.48 ± 0.21 b	6457.57 ± 23.34 b	1460.29 ± 45.44 b	0.71 ± 0.02 b
	S3	52.74 ± 0.10 a	8431.82 ± 15.81 a	1958.84 ± 24.6 a	0.83 ± 0.03 a
	Mean	42.09B	6455.28B	1490.96B	0.71B
N2	S1	31.95 ± 0.40 c	4733.20 ± 11.38 c	1185.23 ± 7.54 c	0.64 ± 0.01 c
	S2	46.70 ± 0.28 b	6885.67 ± 24.89 b	1610.93 ± 2.99 b	0.74 ± 0.03 b
	S3	55.56 ± 0.24 a	8778.74 ± 71.01 a	2180.04 ± 5.60 a	0.86 ± 0.04 a
	Mean	44.74A	6799.20A	1658.73A	0.74A

Note: Different lowercase letters indicate significant differences found using the LSD test for *p* < 0.05 between planting density treatments under the same different nitrogen forms; different uppercase letters indicate significant differences found using the LSD test for *p* < 0.05 between N1 and N2. N1 and N2 represent NO_3_^−^ and NH_4_^+^ fertilizers, respectively. NO_3_^−^ and NH_4_^+^ are provided by calcium nitrate and ammonium sulfate fertilizer, respectively. D1: 30 × 10 cm; D2: 20 × 20 cm; D3: 30 × 20 cm.

## Data Availability

Dataset available on request from the authors. The data are not publicly available due to privacy.
